# *Plasmodium* AdoMetDC/ODC bifunctional enzyme is essential for male sexual stage development and mosquito transmission

**DOI:** 10.1242/bio.016352

**Published:** 2016-07-07

**Authors:** Robert J. Hart, Atif Ghaffar, Shaymaa Abdalal, Benjamin Perrin, Ahmed S. I. Aly

**Affiliations:** Department of Tropical Medicine, Tulane University, New Orleans, LA 70112, USA

**Keywords:** Malaria, Plasmodium, Anopheles, Sexual stages, Polyamines, *S*-adenosyl methionine decarboxylase/ornithine decarboxylase

## Abstract

Polyamines are positively-charged organic molecules that are important for cellular growth and division. Polyamines and their synthesizing enzymes are particularly abundant in rapidly proliferating eukaryotic cells such as parasitic protozoa and cancer cells. Polyamine biosynthesis inhibitors, such as Elfornithine, are now being considered for cancer prevention and have been used effectively against *Trypanosoma brucei*. Inhibitors of polyamine biosynthesis have caused growth arrest of *Plasmodium falciparum* blood stages *in vitro,* but in *P. berghei* only partial inhibition has been observed. While polyamine biosynthesis enzymes are characterized and conserved in *Plasmodium* spp., little is known on the biological roles of these enzymes inside malaria parasite hosts. The bifunctional polyamine biosynthesis enzyme *S*-adenosyl methionine decarboxylase/ornithine decarboxylase (AdoMetDC/ODC) was targeted for deletion in *P. yoelii*. Deletion of AdoMetDC/ODC significantly reduced blood stage parasitemia but *Anopheles* transmission was completely blocked. We showed that male gametocytogenesis and male gamete exflagellation were abolished and consequently no ookinetes or oocyst sporozoites could be generated from *adometdc/odc(–)* parasites. Supplementation of putrescine and spermidine did not rescue the defective phenotypes of male gametocytes and gametes of the knockout parasites. These results highlight the crucial role of polyamine homeostasis in the development and functions of *Plasmodium* erythrocytic stages in the blood and in the mosquito vector and validate polyamine biosynthesis pathway enzymes as drug targeting candidates for malaria parasite transmission blocking.

## Abstract (precis)

**Summary:** We provide the first genetic evidence for the crucial roles of *de novo* polyamine biosynthesis in the development of *Plasmodium* male sexual stages and in the transmission of malaria parasite to mosquito.

## INTRODUCTION

In spite of the reduction of malaria related deaths in sub-Saharan Africa in the last few years, continuous mosquito transmission of *Plasmodium* still poses a tremendous threat to malaria eradication efforts (Alonso et al., 2011). The bias of malaria treatment programs towards chemotherapy approaches targeting asexual pathogenic stages in symptomatic individuals may have contributed to the continuous high transmission rates in endemic areas (Lin et al., 2014). The appearance of artemisinin-resistant strains of *P. falciparum* in Africa and Southeast Asia with high transmission efficiency in different mosquito vectors could contribute to the alarming rapid horizontal and vertical spread of the resistant strains (Fairhurst, 2015; St Laurent et al., 2015). Therefore, more attention is urgently needed towards developing antimalarial treatments that block malaria parasite development in the mosquito and in liver on the community level.

One of the biochemical pathways that has gained attention as a target for antiprotozoal treatment, and more recently as a target for cancer chemoprevention, is the polyamine biosynthesis pathway (Birkholtz et al., 2011; Heby et al., 2003; Pegg, 2009; Wallace, 2009). The three polyamine molecules (the diamine putrescine, the triamine spermidine and the tetramine spermine) are aliphatic positively charged molecules. No specific molecular physiological roles have yet been assigned to polyamines. Nevertheless, they are known to be important for cell growth and division in eukaryotic cells (Birkholtz et al., 2011; Pegg, 2009; Wallace, 2009). One of the drugs that has gained attention because of its ability to cure coma patients infected with African sleeping sickness causative parasite *Trypanosoma brucei* is Elfornithine, or DFMO (α-difluoromethylornithine). Elfornithine is a specific inhibitor for the ODC (ornithine decarboxylase) enzyme of *Trypanosoma brucei gambiense*, with minimal side effects in humans (Birkholtz et al., 2011; Heby et al., 2007, 2003). Elfornithine is now also being considered for cancer chemoprevention (Rial et al., 2009). Elfornithine was used against *P. falciparum* and showed very promising growth inhibiting effects *in vitro* (Assaraf et al., 1984). However, it was not effective against intraerythrocytic stages of the murine malaria model *P. berghei* (Bitonti et al., 1987)*.* Nevertheless, Elfornithine blocked malaria parasite transmission to the mosquito and liver stage development of *P. berghei* (Gillet et al., 1983; Hollingdale et al., 1985).

Intriguingly, *Plasmodium* is the only known living organism that has one open reading frame encoding two enzymes of this pathway, which are *S*-adenosyl methionine decarboxylase (AdoMetDC) and ODC (Krause et al., 2000; Müller et al., 2000). Both decarboxylase domains on the same protein were shown to be functionally and biochemically independent from each other (Wrenger et al., 2001). AdoMetDC converts adenosyl methionine into decarboxylated adenosyl methionine (dcAdoMet) and ODC converts ornithine into the diamine putrescine. This highlights the importance of the temporal regulation of the synthesis of both enzyme products in *Plasmodium* (Birkholtz et al., 2011). Both putrescine and dcAdoMet are obligate substrates for the *de novo* biosynthesis of spermidine by the enzyme spermidine synthase (SpdS) (Pegg, 2009; Wallace, 2009). In *Plasmodium*, SpdS is suggested to be the main enzyme responsible for the biosynthesis of spermine, as the genomes of all species of the malaria parasite lack spermine synthase (SpmS) coding sequence (Birkholtz et al., 2011). In addition to the biosynthesis of polyamines, *Plasmodium* parasites are able to actively salvage polyamines from their hosts, through an unknown transporter (Ramya et al., 2006). Recently, this polyamine transport mechanism was shown to be dependent on the parasite plasma membrane potential (Niemand et al., 2012).

Despite the fact that polyamine-synthesizing enzymes and transport dynamics have been biochemically characterized in *Plasmodium* spp., very little is known about their actual biological functions inside the mammalian host or the mosquito vector. We have evaluated the effect of the absence of the bifunctional enzyme AdoMetDC/ODC on the development of the life cycle stages of the rodent malaria species *P. yoelii* in the mouse and mosquito. Gene deletion studies highlight the need for polyamine *de novo* synthesis for normal growth of asexual stages and for the generation and function of male gametocytes. This is the first described biological function of a polyamine-synthesizing enzyme in *Plasmodium*.

## RESULTS

### Targeted deletion of the malaria parasite conserved AdoMetDC/ODC in *P. yoelii*

**Fig. 1.**
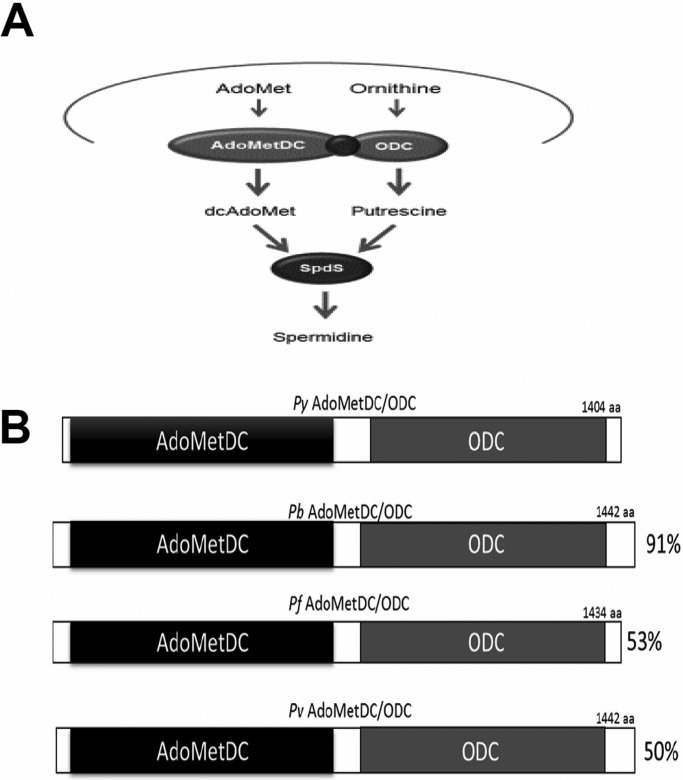
**Conservation of the polyamine biosynthesis pathway in *Plasmodium*.** (A) Schematic representation of the polyamine biosynthesis pathway in *Plasmodium* spp. Ornithine and *S*-adenosyl methionine (AdoMet) are decarboxylated by AdoMetDC/ODC into putrescine and dcAdoMet, which are the substrates for the synthesis of spermidine by the enzyme spermidine synthase (SpdS). It is suggested that spermine is also synthesized by SpdS using spermidine and dcAdoMet as substrates. (B) Schematic representation of conservation of the bifunctional enzyme AdoMetDC/ODC in rodent and human malaria parasite species. Whole protein amino acid identity compared to *Py*AdoMetDC/ODC is shown on the left of the bars representing the proteins.

**Fig. 2.**
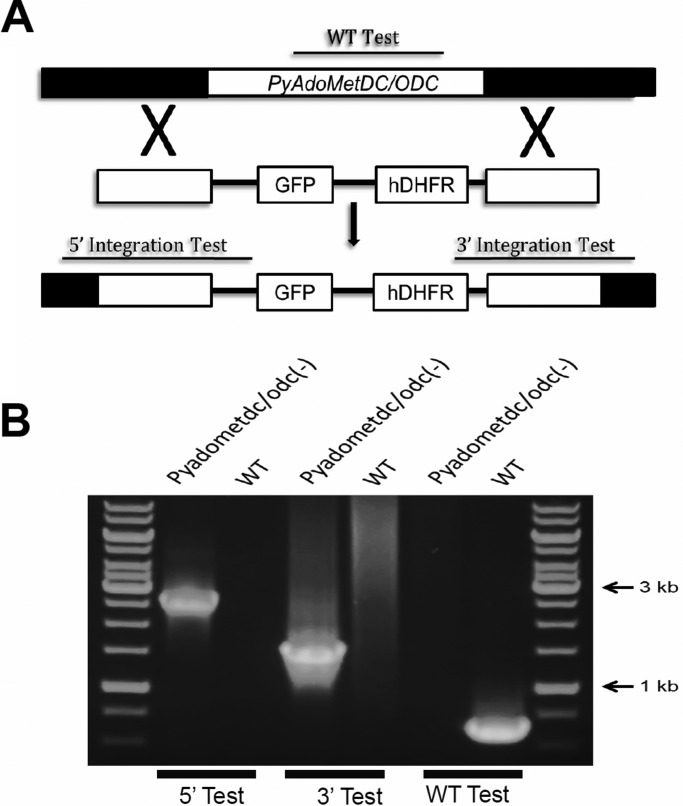
**Targeted deletion of AdoMetDC/ODC in *P. yoelii*.** (A) Schematic representation of the replacement strategy to generate *Pyadometdc/odc(–)* parasites. The endogenous *Py*AdoMetDC/ODC genomic locus is targeted with homologous replacement fragments containing upstream and downstream sequences of *PyAdoMetDC/ODC* flanking the human DHFR positive selection marker and eGFP cassettes. The positions of WT-specific (WT Test) or integration-specific (5′ Test and 3′ Test) test amplicons are indicated by lines. (B) Diagnostic 36-cycle PCR genotyping confirms the integration of gene-replacement construct using oligonucleotide primer combinations that can only amplify from the recombinant locus (5′ Test and 3′ Test). The WT-specific PCR reaction (WT Test) confirms the absence of WT parasites in *Pyadometdc/odc(–).* The arrows show the size of DNA ladder bands of 3000 and 1000 bps, respectively.

Apart from the absence of a clear homologue for spermine synthase, all of the polyamine biosynthesis enzymes are conserved in *Plasmodium* genomes (Fig. 1A). The first two enzymes, AdoMetDC and ODC, which are present on the same open reading frame, are highly conserved in all *Plasmodium* species with an overall amino acid identify of more than 50% between rodent and human malaria parasite species, with a much higher degree of amino acid identity in both enzymatic domains (Fig. 1B). In order to determine the role of the bifunctional enzyme AdoMetDC/ODC in parasite development in host erythrocytes and in transmission to the mosquito, we employed a reverse genetics approach to target *AdoMetDC/ODC* in the rodent malaria model *P. yoelii* 17XNL non-lethal strain (*Py*WT). To create a single gene deletion by a double cross-over homologous recombination (Fig. 2A), the 5′ and 3′UTR regions of *PyAdoMetDC/ODC* (PlasmoDB ID: PY17X_0518000) were cloned into the targeting vector pAA20 (Hart et al., 2014). As a control for the transfection strategy and procedures, we applied the same strategy to target the *P. yoelii P230p* gene, which has previously been shown in our lab and numerous other labs to be dispensable for all malaria parasite life cycle stages (Hart et al., 2014; Janse et al., 2006; Lin et al., 2011; Manzoni et al., 2014; van Dijk et al., 2010). Transfection with the targeting vectors, drug selection, cloning of transgenic parasites and genomic PCR analysis were performed, as previously described (Hart et al., 2014), to confirm the deletion of *PyAdoMetDC/ODC* (Fig. 2B). The ability to knockout *PyAdoMetDC/ODC* demonstrates that the *de novo* biosynthesis of the polyamine putrescine and dcAdoMet (the other substrate for spermidine biosynthesis) is not essential for survival of asexual blood stage parasites of *P. yoelii*. Of note, as we were initially expecting *PyAdoMetDC/ODC* to be essential for blood stage parasites, and therefore refractory for disruption, we transfected a knock-in construct to replace *PyAdoMetDC/ODC* with a functional copy of itself, to evaluate the accessibility of the gene locus (data not shown). Both the knockout and knock-in were transfected at the same time from the same donor transfection culture. The clones of *PyAdoMetDC/ODC knock-in (*KN*)* were very comparable to the development of wild-type (WT) or WT-like *Pyp230p(–)* parasites in all malaria parasite life cycle stages tested (Data not shown). Both *Pyadometdc/odc(–)* and *Pyp230p(–)* also expressed the fluorescent marker eGFP (enhanced GFP) as expected (Fig. S1).

### AdoMetDC/ODC is required for normal *Plasmodium* blood stage growth

**Fig. 3.**
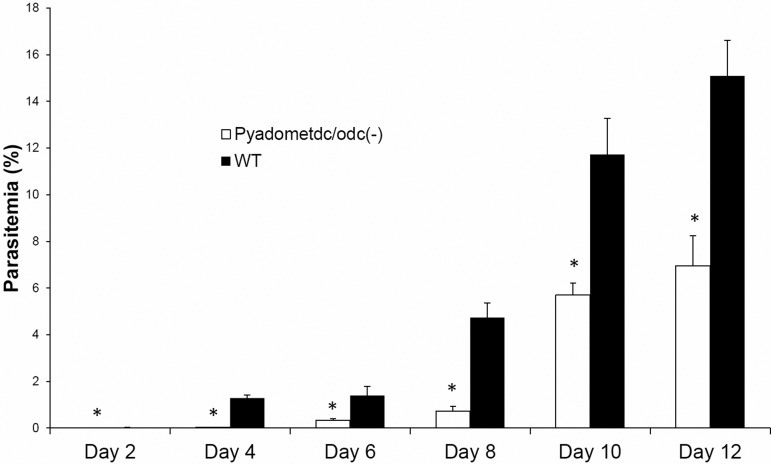
**Blood stage growth deficiency of *Pyadometdc/odc(–)* parasites.** Average blood stage parasitemia (evaluated as percentage of infected erythrocytes in at least 50 grid microscopic fields) in groups of four BALB/c mice per genotype (not pre-treated with phenylhydrazine) after IV injection of 5000 infected erythrocytes per mouse with *Pyadometdc/odc(–)* or *Py*WT 17XNL parasite strains. The graph shows significantly reduced parasitemia (denoted by an asterisk) of *Pyadometdc/odc(–)* compared to WT in all days tested (Day 2, *P=0.0039; day 4-12, *P<0.0001). Data are represented as mean±s.d.

To assess the importance of *Py*AdoMetDC/ODC during the intraerythrocytic phase of *P. yoelii* development, we compared blood stage parasitemia between two groups of BALB/c mice intravenously (IV) infected with 5000 blood stage parasites of *Pyadometdc/odc(–)* and WT 17XNL parasites. Parasitemia of all infected mice were recorded from Giemsa-stained thin blood smears until clearance at day 17 or 18 post-infection (PI). We used a *t*-test to determine significance in all experiments listed with significance set at *P* value of <0.05. Significant reduction in blood stage parasitemia was detected on all days recorded for *Pyadometdc/odc(–)* parasites (day 2, *P*=0.0039; days 4-12, *P*<0.0001; Fig. 3). Despite this significant reduction in parasitemia of parasites lacking *Py*AdoMetDC/ODC, no morphological differences were observed in *Pyadometdc/odc(–)* parasites compared to WT or WT-like parasites (Fig. S2). These findings demonstrate that *PyAdoMetDC/ODC* is important, but not essential, for blood stage growth in the mammalian host.

### AdoMetDC/ODC is essential for male gametocytogenesis and male gamete exflagellation

**Fig. 4.**
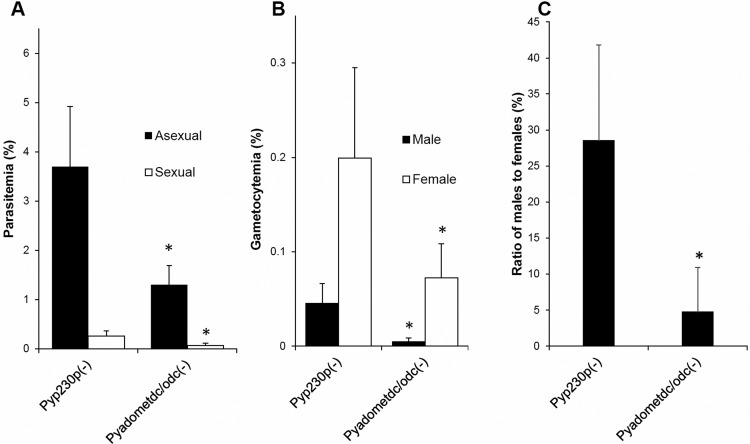
**Male gametocytogenesis deficiency in *Pyadometdc/odc(–)* parasites.** (A) Average blood stage parasitemia (evaluated as percentage of infected erythrocytes) for asexual and sexual stages of *Pyadometdc/odc(–)* and *Pyp230p(–)* parasites in groups of three Swiss Webster mice per genotype (pre-treated with 50 mg/kg phenylhydrazine). Both asexual and sexual stage parasitemia of *Pyadometdc/odc(–)* parasites are significantly reduced compared to WT-like *Pyp230p(–)* parasites (Asexual, *P<0.0001; Sexual, *P<0.0006). (B) Graph shows the average percentage of mature male and female gametocytes. Only male gametocytogenesis of *Pyadometdc/odc(–)* is significantly reduced compared to *Pyp230p(–)* parasites (Male, *P=0.0024; Female, *P=0.0048). (C) The sexual ratio of mature male gametocytes to female gametocytes, which confirms the observation that male gametocytogenesis is specifically deficient in *Pyadometdc/odc(–)* parasites compared to *Pyp230p(–)* parasites (*P=0.021334). The results shown in all graphs are the averages of at least three independent experiments. Three mice per genotype are used in each independent experiment. The statistical significant deficiencies in *Pyadometdc/odc(–)* parasites are denoted by an asterisk. Data are represented as mean±s.d.

The availability of *Pyadometdc/odc(–)* blood stage parasites made it possible to assess sexual stages development and transmission to the mosquito vector. Outbred Swiss Webster mice pre-treated with phenylhydrazine, to enhance gametocytogenesis (Ramakrishnan et al., 2013), were IV infected with 1×10^6^ of *Pyadometdc/odc(–)* and *Pyp230p(–)* blood stage parasites. At day 3 PI, thin blood smears were Giemsa-stained to estimate the asexual and sexual blood stage parasitemia and the percentages of female and male gametocytes of both transgenic parasite strains in infected mice. There was a significant reduction in parasitemia percentage for both sexual and asexual stages in *Pyadometdc/odc(–)* compared to *Pyp230p(–)* parasites (Sexual, *P*<0.0006; Asexual, *P*<0.0001; Fig. 4A). Moreover, the evaluation of the percentage of male and female gametocytemia defined a clear significant reduction for both male and female gametocytes in *Pyadometdc/odc(–)* (Male, *P*=0.0024; Female, *P*=0.0048; Fig. 4B). However, when we compared the ratios of male to female gametocytes, we noticed that the ratio of *Pyadometdc/odc(–)* males to females was consistently less than half of the male to female ratio for *Pyp230p(–)* (*P*=0.021334; Fig. 4C)*.* This indicates that the lack of *Py*AdoMetDC/ODC did not lead to an equal reduction in all sexual blood stages but there was a specific negative impact on the gametocytogenesis of male gametocytes.

**Fig. 5.**
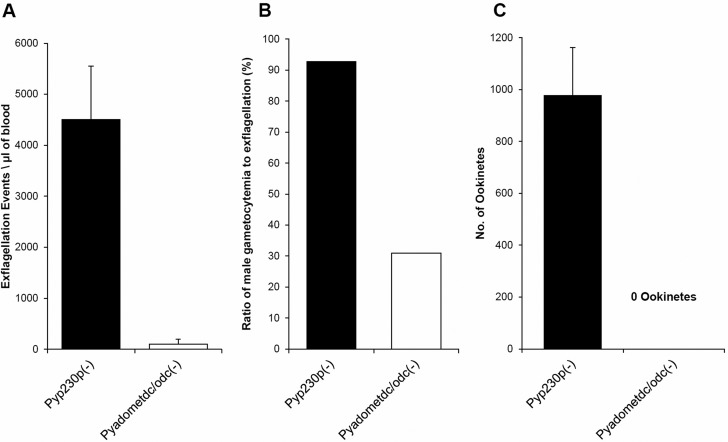
**Parasites lacking AdoMetDC/ODC are deficient in male gamete exflagellation and transmission to the mosquito.** (A) Graph shows the average number of male gamete exflagellation events per µl of mouse blood determined by a hemocytometer using 1:10 dilution of tail blood of *Pyadometdc/odc(–)* compared to WT-like *Pyp230p(–)* parasites. The formation of male gametes is almost completely absent in the knockout parasites (*P*<0.001). (B) Graph shows the male gametocyte exflagellation efficiency ratio, which confirms the observation that the inhibition of exflagellation in *Pyadometdc/odc(–)* parasites is due to a deficiency in male gametogenesis rather than reduced numbers of male gametocytes. (C) Graph shows average number of ookinetes dissected out of mosquitoes infected with *Pyadometdc/odc(–)* compared to *Pyp230p(–)* parasites 20 h pmf. The results shown in all graphs are the averages of at least three independent experiments. Three mice per genotype (pre-treated with 50 mg/kg phenylhydrazine) are used in each independent experiment. Data are represented as mean±s.d.

Next, we evaluated the effect of the absence of *Py*AdoMetDC/ODC on the formation of male gametes (gametogenesis), which is determined by the quantification of male gamete exflagellation. To quantify average male gamete exflagellation per µl of infected blood, we collected blood from both infected mouse groups at day 3 PI. Exflagellation of male gametes of *Pyadometdc/odc(–)* was almost abolished compared to the potent exflagellation of *Pyp230p(–)* male gametes (*P*<0.001; Fig. 5A). In order to differentiate between a severe exflagellation deficiency simply due to the reduced percentage of male gametocytes and a deficiency in gametogenesis, we evaluated the male gametocyte exflagellation efficiency ratio, which is the percentage of male gametocytes to male gametes. Whereas the male gametocyte exflagellation efficiency ratio was over 90% for *Pyp230p(–)* parasites, the ratio was about 30% for *Pyadometdc/odc(–)* male gametocytes (Fig. 5B). Therefore, the abolished male gametogenesis was not just due to the reduced numbers of male gametocytes that were exflagellating but also due to a deficiency in male gametogenesis. Collectively, these data provide clear evidence that AdoMetDC/ODC plays a crucial role for male gametocytogenesis and gametogenesis of the malaria parasite.

### Transmission to the mosquito is completely blocked in parasites lacking AdoMetDC/ODC

To examine the importance of *Py*AdoMetDC/ODC for the development of ookinetes and oocyst sporozoites, *Anopheles stephensi* mosquitoes were fed on the infected mice that showed the highest male gamete exflagellation rate. After 20 h pmf (post-mosquito feeding), female mosquitoes midguts were dissected and mature ookinetes were counted for both parasite genotype, as previously described (Hart et al., 2014). A complete abolishment of ookinete formation was observed in the knockout parasite compared to WT-like controls (Fig. 5C). Furthermore, mosquito midguts were dissected and ground to release and count oocyst sporozoites at day 10 pmf, as previously described (Aly et al., 2010; Hart et al., 2014). Not a single oocyst sporozoite was detected in the midguts of female mosquitoes infected with *Pyadometdc/odc(–)* parasites in three independent experiments (Table 1). This indicates that transmission to the mosquito was completely blocked in parasites lacking *Py*AdoMetDC/ODC bifunctional enzyme.

**Table 1.**
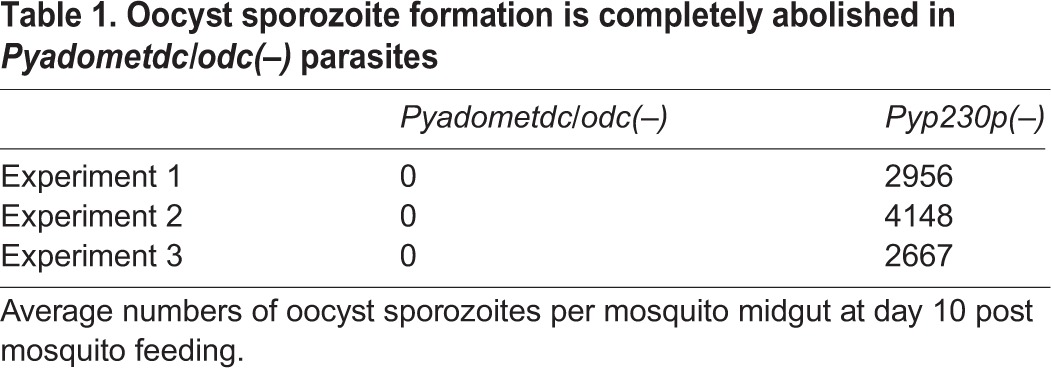
**Oocyst sporozoite formation is completely abolished in *Pyadometdc/odc(–)* parasites**

### Polyamine supplementation did not rescue the defective male sexual stages development and exflagellation in AdoMetDC/ODC deficient parasites

**Fig. 6.**
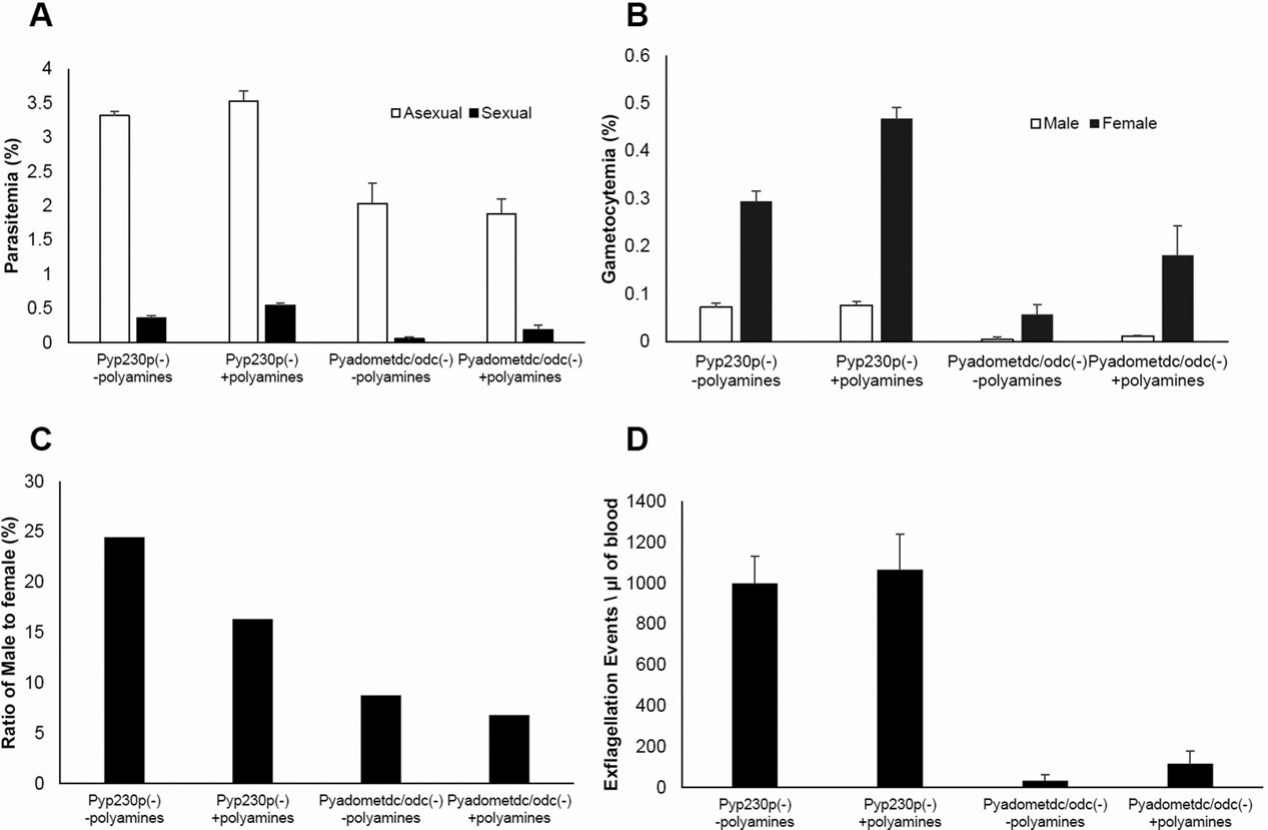
**Putrescine and spermidine supplementation partially increase male gametocytogenesis and gametogenesis but do not rescue the defective phenotypes of *Pyadometdc/odc(–)*.** (A) Graph shows average blood stage parasitemia for asexual and sexual stages of *Pyadometdc/odc(–)* and *Pyp230p(–)* parasites in groups of three Swiss Webster mice per genotype with (+polyamines) or without (−polyamines) the supplementation of polyamines mixture of putrescine and spermidine (100 mg/kg for each polyamine). The supplementation of polyamines did not rescue the defective asexual and sexual blood stage growth of *Pyadometdc/odc(–)* compared to *Pyp230p(–)* parasites. (B) Graph shows average percentage of mature male and female gametocytes of *Pyadometdc/odc(–)* compared to *Pyp230p(–)* parasites with or without the supplementation of polyamines. The deficiency in male gametocytogenesis phenotype of *Pyadometdc/odc(–)* parasites was not rescued by the supplementation of polyamines. There was an increase in female gametocytogenesis that was observed in both *Pyadometdc/odc(–)* and *Pyp230p(–)* parasites after the supplementation of polyamines. (C) Graph shows ratio of mature male to female gametocytes of *Pyadometdc/odc(–)* compared to *Pyp230p(–)* parasites with or without the supplementation of polyamines. The diminished ratios of male to female gametocytes in the knockout parasites after polyamine supplementation confirm that that the deficiency in male gametocyte formation is not associated with the overall reduction in the formation of blood stage parasites. (D) Graph shows average number of male gamete exflagellation events per µl of mouse blood determined by a hemocytometer using 1:10 dilution of tail blood of *Pyadometdc/odc(–)* compared to *Pyp230p(–)* parasites with or without the supplementation of polyamines. Polyamine mixture was added to the diluted blood exflagellation samples (0.5 mg/ml) from mice that were treated with polyamine mixture injection. The formation of male gametes is almost completely absent in the knockout parasites even after polyamine supplementation. There was an improved male gametocytogenesis in B and male gametogenesis in D in *Pyadometdc/odc(–)* parasites that were supplemented with polyamines compared to the *Pyadometdc/odc(–)* parasites that were supplemented with PBS. This provides evidence that defective phenotypes of the knockout parasites were due to a deficiency in polyamine *de novo* biosynthesis that could not be fully rescued by the supplementation of high concentrations of polyamines. Data are represented as mean±s.d.

To examine the ability of polyamine supplementation to rescue the developmental deficiencies in blood stage development, male gametocyte development and male gamete exflagellation in *Pyadometdc/odc(–)* parasites, we injected mice that had been first IV infected with 1×10^6^ blood stage parasites of *Pyadometdc/odc(–)* and *Pyp230p(–)* with four injections of putrescine and spermidine mixture, at a concentration of 100 mg/kg IP (intraperitoneal) for each polyamine, for three consecutive days starting at the day of parasite infection. Each group of polyamine-supplemented mice had a parallel control infected mouse group that received only injections of PBS. At day 3 PI, we evaluated the parasitemia, gametocytemia and male gamete exflagellation for all mouse groups. No growth rescue of the reduced asexual and sexual male and female blood stage growth of AdoMetDC/ODC-deficient parasites was observed after supplementation with polyamines compared to the *Pyp230p(–)* infected control mouse groups with or without the supplementation of polyamines (Fig. 6A,B). Moreover, male to female gametocyte ratio for *Pyadometdc/odc(–)* was still significantly reduced after polyamines supplementation compared to the ratios of male to female gametocytes of *Pyp230p(–)*-infected control mouse groups with or without the supplementation of polyamines (Fig. 6C). For the male gamete formation assay, we added putrescine and spermidine mixtures, at a concentration of 0.5 mg/ml to the 1:10 diluted blood sample before loading the sample on a hemocytometer for counting of exflagellation events. While the control *Pyp230p(–)* blood stage parasites displayed a high male gamete exflagellation rate with or without the supplementation of polyamines to infected mice and to the exflagellation sample, the knockout *Pyadometdc/odc(–)* did not show any significant increase in exflagellation rate with the supplementation of polyamines compared to the WT-like control parasites with or without polyamines supplementation (Fig. 6D). Since the polyamines putrescine and spermidine can be efficiently transported into the intraerythrocytic malaria parasite (Niemand et al., 2012), these data provide evidence that the deficiencies observed in asexual and sexual stages growth and male gamete exflagellation are due to the inability to *de novo* synthesize putrescine and dcAdoMet and therefore possibly also spermidine. Of note, the supplementation of polyamines caused the female gametocyte numbers to increase in both the knockout and control parasite infected groups compared to the knockout and control parasite infected groups without polyamine supplementation, respectively (Fig. 6B). Moreover, we also observed a small increase in the male gamete exflagellation rate in the knockout *Pyadometdc/odc(–)* after polyamine supplementation compared to the *Pyadometdc/odc(–)* group without polyamine supplementation (Fig. 6D). These observations may indicate that polyamine supplementation, though not capable of fully complementing the defective phenotypes, leads to a partial rescue, possibly through the increased transport into the parasites. Collectively, the supplementation experiments results clearly confirm an essential role of the polyamine *de novo* synthesis in the growth and development of asexual and sexual parasites in the blood and transmission to the mosquito vector.

## DISCUSSION

Alongside the intraerythrocytic asexually replicating parasites, intrinsic and extrinsic factors lead to the development of sexual stages that are only destined for transmission and continuation of the life cycle (Waters, 2016). These complex and variable factors are not yet fully understood. Nonetheless, it is believed that the development of sexual stages involves signaling cascades, which activate epigenetic chromatin changes to induce large-scale transcriptional regulation of genes (Waters, 2016). The plant-like transcription factor Api AP2-G was shown to be involved in the upregulation of genes that are responsible for the gametocytogenesis or the induction of gametocytes (Kafsack et al., 2014; Sinha et al., 2014). The fact that asexual and sexual stages were both reduced in AdoMetDC/ODC knockout parasites does not suggest that polyamines are involved in the transcriptional regulation of gametocytogenesis but rather have an overall cellular effect on the rate of growth and replication in blood stage parasites. However, we showed that the reduction in male gametocytes is not due to a significant decrease in blood stage parasite burden, but rather to a severe defect in male, and not female, gametocytogenesis. An explanation for the specific deficiency to form male gametocytes (due to a cellular but not a transcriptional cause) is the failure to differentiate into mature male gametocytes rather than a deficiency in inducing microgametocytogenesis. While polyamines could be salvaged to the parasite in the blood (Niemand et al., 2012; Ramya et al., 2006), the *de novo* synthesis is needed for efficient growth and development of asexual and sexual stages and specifically for the differentiation of male gametocytes. Nonetheless, the transport of polyamines could account for the small number of male gametocytes that were formed in the knockout parasites. The availability of more polyamines due to the exogenous supplementation improved the rate of microgametocytogenesis but was not sufficient to rescue the defective phenotypes.

Following the completion of gametocytogenesis, fully differentiated male and female gametocytes roam the peripheral blood circulation (Aly et al., 2009). Upon ingestion of *Plasmodium* infected blood by *Anopheles* female mosquito, gametogenesis is initiated in the mosquito midgut. The microgametocyte is activated to replicate by mitosis and conclude cytokinesis in less than 10 min to produce up to eight motile male microgametes (Aly et al., 2009). Our results confirmed that male gametogenesis was almost completely blocked in *Pyadometdc/odc(–)* specifically due to a male gamete deficiency. This can only be explained by the necessity of actively *de novo* synthesized polyamines during this process that has to be accomplished within few minutes, with a possible role of the newly synthesized polyamines in mobilizing DNA during the rapid cytokinesis. This indicates that the polyamine transport mechanisms (Niemand et al., 2012; Ramya et al., 2006), which possibly partially rescued intraerythrocytic growth in the blood, was not sufficient to sustain the abrupt need for polyamines in a short period of time during gametogenesis. There was some evidence that male gamete exflagellation increased with polyamine supplementation, but not enough to rescue it to the exflagellation rate of the *Pyp230p(–)* knockout within the few minutes allotted. It is also important to note that Niemand et al. (2012) and Ramya et al. (2006) only studied the polyamine transport mechanisms in asexual blood stage parasites, but these transport mechanisms have not yet been characterized in sexual blood stages. Therefore, based on our results, research into inhibition of polyamine transport in sexual blood stage parasites could prove valuable (Reguera et al., 2005). These observations indicate that polyamines could be implicated in multiple functions during the intraerythrocytic development of *Plasmodium,* most notably in male gametocytogenesis and gametogenesis.

Collectively, the combined deficiencies in male gametocytogenesis and gametogenesis resulted in no ookinete formation and consequently no oocyst or oocyst sporozoite formation and thus *Plasmodium* mosquito transmission was completely blocked. These results confirm earlier studies that showed blocked transmission after treatment of *P. berghei* infected mice with polyamine inhibitors (Gillet et al., 1983). The almost complete inhibition of male gametogenesis did not allow for evaluation of the effect of *Py*AdoMetDC/ODC absence on other mosquito stages or on liver stage development. Therefore, polyamine biosynthesis may be essential for other life cycle stages of the malaria parasite as earlier studies have indicated a potent blockage of *P. berghei* liver stage development after treatment with polyamine biosynthesis inhibitors (Hollingdale et al., 1985). This highlights the importance of this pathway as a potent drug target for antimalarials that could block mosquito and pre-erythrocytic stage development. In addition, biosynthesis of spermidine and spermine may present potent targets against malaria parasite blood stage parasites, with some preliminary evidence for antimalarial activity of spermidine synthase inhibitors (Burger et al., 2015; Sprenger et al., 2015). Fortunately, the attention that this pathway has recently gained as a cancer prevention target allowed for the generation of effective polyamine biosynthesis inhibitors with lower human toxicity that may also be used as antimicrobial drugs against protozoan parasites.

## MATERIALS AND METHODS

### Experimental animals, parasites and mosquitoes

Mice and mosquitoes were infected with wild-type (WT) *P. yoelii* 17XNL Clone 1.1 (non-lethal strain), WT-like *Pyp230p(–)* clone A5 (Hart et al., 2014), *Pyadometdc/odc(–)* clones K1 and K2 and *PyAdoMetDC/ODC* knock-in (KN) clone M1 parasites as previously described (Hart et al., 2014). Animal handling was conducted according to Institutional Animal Care and Use Committee (IACUC)-approved protocols.

### Generation of transgenic parasites

Targeted deletions of *PyAdoMetDC/ODC* and *PyP230p* genes were accomplished by double crossover homologous recombination, using the same transfection strategy and the same transfection vector, as previously described (Hart et al., 2014). The generation of *Pyp230p(–)* clone A5 parasites and their phenotypic analyses compared to WT are described in a previous study (Hart et al., 2014). To generate the transfection knockout plasmid for *PyAdoMetDC/ODC* genomic locus, DNA fragments of the 5′UTR and the 3′UTR of *PyAdoMetDC/ODC* were amplified from *P. yoelii* 17XNL genomic DNA (gDNA) using primer pairs 71-72 and 73-74, respectively (Table 2), and the amplified fragments were inserted into the transfection plasmid AA20 between *Sac*II-*Bam*HI and *Kpn*I-*Hind*III restriction enzyme sites, respectively. The final plasmid was linearized with *Sac*II and *Kpn*I prior to the transfection. Transfection, drug selection and parasite cloning was done as previously described (Aly et al., 2010; Hart et al., 2014). 5′ Integration Tests were performed with primers 75-16 and 3′ Integration Tests with primers 17-76 and WT coding sequence tests with primers 77-78 (Table 2).

**Table 2.**
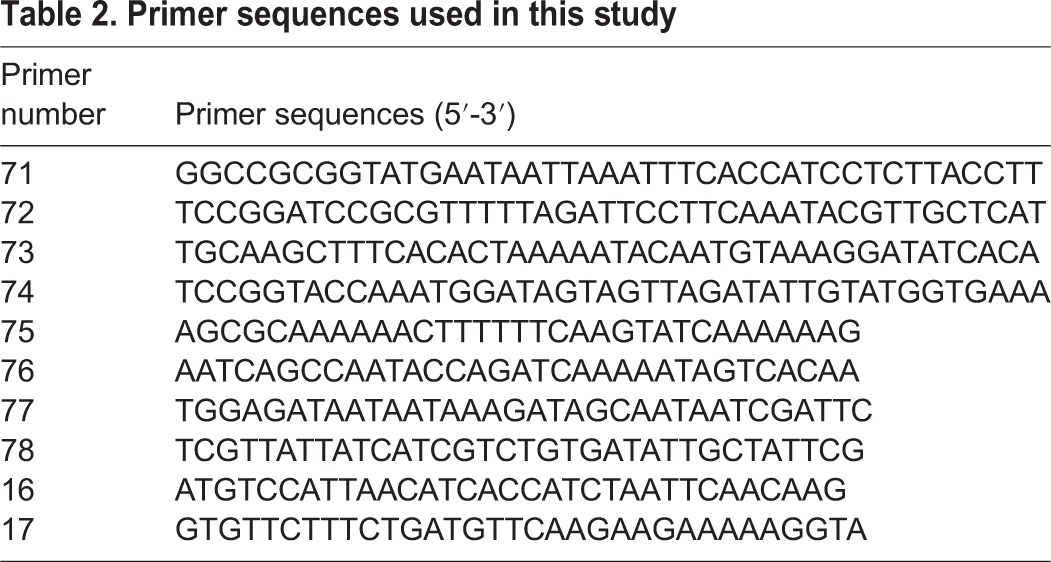
**Primer sequences used in this study**

### Phenotypic analysis of asexual and sexual blood stage parasites

These experiments were done as previously described (Hart et al., 2014).

### Polyamine supplementation experiments

All the mice used in the polyamine supplementation experiments were not pre-treated with phenylhydrazine as the mice used in the similar initial phenotypic analysis experiments. Groups of three mice were injected with a mixture of the polyamines putrescine and spermidine (purchased from Sigma), each at a concentration of 100 mg/kg, dissolved in PBS. Each mouse received four IP injections of 100 µl of the polyamine mixture starting at the day of parasite IV infection and subsequently at days 1, 2 and 3 PI thereafter. Parallel control groups of mice received injections of PBS starting at the day of parasite IV infections and at the three days thereafter. All phenotypic analyses of blood stage growth, gametocyte ratio estimation and male gamete exflagellation were done as previously described (Hart et al., 2014).

### Statistical analysis

A *t*-test was used to assess the median values of all three genotypes for statistically significant differences in all experiments. GraphPad InStat software was used for all analysis. *P*-values of <0.05 were considered statistically significant.
